# Characteristics of peritoneal dialysis-related infection according to pet ownership: An 8-year single-center experience

**DOI:** 10.1371/journal.pone.0348012

**Published:** 2026-05-07

**Authors:** Hyeon Gyu Cho, Jeong Geon Lee, Jung Wan Park, Jihee Lim, Nam Hun Heo, Nam-Jun Cho, Samel Park, Hyo-Wook Gil, Eun Young Lee

**Affiliations:** 1 Department of Internal Medicine, Soonchunhyang University Cheonan Hospital, Cheonan, Korea; 2 Department of Medicine, Soonchunhyang University College of Medicine, Cheonan, Korea; 3 Department of Biostatistics, Soonchunhyang University Cheonan Hospital, Cheonan, Korea; Japanese Red Cross Medical Center, JAPAN

## Abstract

With rising pet ownership, concerns regarding pet-related infections during peritoneal dialysis (PD) have increased. This retrospective study analyzed the characteristics of PD-related infections according to pet ownership. A total of 162 PD patients treated at Soonchunhyang University Cheonan Hospital between 2016 and 2023 were reviewed. Patients were grouped by pet ownership and pet type (dog or cat) based on data obtained from PD nurse home visits. Peritonitis, exit-site infection (ESI), and tunnel infection (TI) were defined according to the International Society of Peritoneal Dialysis guidelines, and data on causative organisms and clinical outcomes were collected. Zoonotic microorganism-associated peritonitis episodes were identified in patients with pets. Staphylococcus-associated ESIs (55.6 vs. 16.2%, p = 0.006) were more frequently observed in patients with pets than in those without pets. However, the overall incidence of peritonitis and ESI did not differ significantly between patients with and without pets, and pet ownership was not associated with mortality or PD catheter removal. These findings suggest that while pet ownership may influence the microbial characteristics of PD-related infections, it does not appear to increase overall infection incidence or adverse clinical outcomes.

## Introduction

Patients with end-stage kidney disease (ESKD), the final stage of chronic kidney disease, require kidney replacement therapies such as hemodialysis (HD), peritoneal dialysis (PD), or kidney transplantation to replace essential kidney functions [1]. Among them, PD is a process that involves inserting a catheter into the peritoneal cavity to filter fluids and waste products [2]. One of the main advantages of PD is that patients themselves can perform the procedure, thus making it preferred by some ESKD patients due to its convenience and enhanced mobility. However, the procedure is associated with a risk of infection, and the source of the infection is not only the vulnerable catheter structure but also the patients with kidney failure who have compromised immunity [3,4].

PD is a technique that needs the patient or a caregiver to be in a condition to carry out the process independently. Therefore, it may not be suitable for all ESKD patients [5]. Proper catheter management, particularly maintaining sterility, is important. Contamination during the PD exchange procedure must be avoided since it can lead to PD-related infections such as peritonitis, exit-site infection (ESI), and tunnel infection (TI), which affect patient survival and long-term treatment success [6,7].

Over the past few years, pet ownership has been on the rise worldwide, and South Korea is no exception to the trend despite the country’s decreasing birth rates. For example, the dog population in South Korea increased by 44.6% between 2019 and 2022, and more than 3 million dogs were registered in the country within this period [8]. The number of PD patients who own pets is also increasing, and about 30% of the PD households have pets in the global Peritoneal Dialysis Outcomes and Practice Patterns Study (PDOPPS) [9].

Although there is limited research on whether or not pet ownership is associated with peritoneal dialysis-related infections, the most recent International Society for Peritoneal Dialysis (ISPD) peritonitis guidelines specifically warn of contamination from indoor pets[6]. There were cases of bacterial peritonitis events that were caused by zoonotic organisms from pets through animal bites or scratches [10–13]. Nevertheless, the question of whether pet ownership is associated with PD-related infections remains open since there is a study that found that the presence of pets does not lead to an increased risk of peritonitis, except for patients who have cats and dogs [9].

More studies are needed to determine the potential of pets in the contribution to infections due to zoonotic organisms and the long-term impacts on PD patients. Therefore, our study aims to investigate the characteristics of PD-related infections according to pet ownership status, including incidence, causative organisms, and clinical outcomes.

## Materials and methods

### Study population

**Fig 1:**
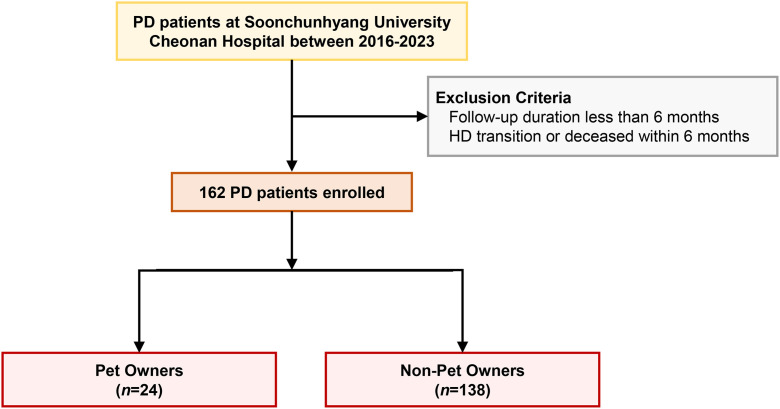
Flow chart of the study.

This study was a retrospective single-center cohort study conducted at Soonchunhyang University Cheonan Hospital, involving patients on PD from January 2016 to December 2023. We included patients with a follow-up period exceeding 6 months, as well as those who transitioned to HD or deceased within 6 months (Fig 1). The study was approved by the Institutional Review Board (IRB) of Soonchunhyang University Cheonan Hospital (IRB No. 2023-07-049) and conducted in accordance with the Declaration of Helsinki of the World Medical Association. The IRB waived the need for informed consent from participants due to the retrospective nature of the study and the use of de-identified data.

### Clinical data collection

The medical records of the study subjects were reviewed to collect demographic information such as age, gender, duration of dialysis, body mass index (BMI), pet ownership, PD modality, and the etiological cause of ESKD. Data were accessed for research purposes from 03/04/2024 to 10/12/2025. Age was based on the time of initial enrollment, and pet ownership was confirmed based on the observations of PD nurses during home visits. Additionally, laboratory test results recorded at the initial time point during the observation period, including hemoglobin, glucose, albumin, total cholesterol, triglycerides, potassium, phosphorus, total calcium, C-reactive protein, and 24-hour urine volume, were also gathered. Furthermore, events of peritonitis, ESI, and TI occurring during the observation period were documented, including details on the causative organisms, occurrence of catheter removal, and mortality. Detailed data underlying these events are provided in S10 and S11 Tables in S1 File in the Supporting Information.

When multiple organisms were isolated from ESIs, the primary pathogen was specifically selected as multiple isolates are common in catheter-related infections near the skin [14]. The criteria for identifying the primary pathogen of ESI were based on the organism that appeared most frequently and in the highest quantities in repeated tests conducted during the same period.

In our center, patients undergoing PD were routinely educated regarding pet-related precautions. They were advised to keep pets separate from the dialysis environment as a general measure, and specifically to avoid close proximity to pets during dialysis exchanges. Exit-site care was performed in accordance with the recommendations of the ISPD, including routine exit-site cleansing and standard prophylactic measures [7].

### Outcomes

The diagnostic criteria for peritonitis, ESI, and TI adhered to the most recent ISPD guidelines [6,7]. According to these guidelines, a definitive exit-site infection is characterized by the presence of purulent discharge. Therefore, catheter-related events without discharge, but with other inflammatory signs such as erythema, tenderness, or swelling, were not included as infection events. Additionally, relapsing peritonitis, ESI, and TI are excluded from the infection events, as they are considered extensions of the original episode. Relapsing infection events were defined as infections occurring within 4 weeks of completing therapy for a prior episode with the same organism or culture-negative episode.

### Statistical analysis

Statistical analyses were performed using SPSS version 25.0. Categorical variables such as gender, pet ownership, and the etiologic cause of ESKD were expressed as counts (percentages). Continuous variables such as duration of dialysis, BMI, and laboratory parameters were presented as mean ± standard deviation or median (interquartile range), as appropriate. For comparisons between two groups, the chi-square test or Fisher’s exact test was used for categorical variables, while the Mann-Whitney U test was employed for continuous variables. Incidence rate was defined as events per person-year, with 95% confidence interval (CI) calculated by the mid-P exact test. Missing data were excluded without imputation.

## Results

### Baseline characteristics

Baseline characteristics of pet owners and non-pet owners are present in Table 1, with a breakdown by pet types detailed in S1 Table in S1 File. Among the 162 patients, 24 patients raised pets, including 8 cat owners and 16 dog owners. The specific number of pets and whether they were kept indoors or outdoors are presented in S2 Table in S1 File. No significant differences in clinical features were observed between pet owners and non-pet owners. The median follow-up duration was 3.00 years (0.94–4.98) for cat owners, 2.67 years (1.09–4.22) for dog owners, and 3.05 years (1.23–5.72) for patients without pets.

**Table 1:** Baseline characteristics of patients according to pet ownership status.

Variables	Pet Owners (*n* = 24)	Non-Pet Owners (*n* = 138)	P value
Demographics			
Age (years)	47.6 (37.9-55.9)	52.9 (44.4-61.9)	0.109
Male sex (%)	41.7	55.8	0.200
Dialysis vintage (month)	153.8 (83.4-185.6)	135.3 (64.1-247.2)	0.992
BMI (kg/m^2^)	25.0 (21.7-30.2)	24.5 (21.5-27.4)	0.445
PD type			
CAPD (%)	54.2	58.7	0.844
APD (%)	45.8	39.1	
CAPD + APD (%)	0.0	1.4	
Hybrid (%)	0.0	0.7	
Primary disease			
Diabetes Mellitus (%)	29.2	47.8	0.064
Hypertension (%)	37.5	34.1	
Chronic Glomerulonephritis (%)	33.3	12.3	
Others (%)	0.0	1.4	
Unknown (%)	0.0	4.3	
Laboratory test			
Hemoglobin (g/dL)	10.2 (9.5-11.1)	10.2 (9.2-11.1)	0.841
Glucose (mg/dL)	103.5 (92.3-116.8)	109.5 (93.0-142.3)	0.177
Albumin (g/dL)	3.9 (3.6-4.2)	3.8 (3.4-4.1)	0.209
Total cholesterol (mg/dL)	152.0 (108.8-192.8)	146.0 (119.8-173.0)	0.055
Triglycerides (mg/dL)	136.5 (135.0-141.0)	124.0 (85.8-177.8)	0.102
Potassium (mEq/L)	4.5 (4.1-4.8)	4.5 (3.9-4.9)	0.934
Phosphorus (mg/dL)	5.2 (4.2-6.4)	4.9 (4.0-5.8)	0.249
Total calcium (mg/dL)	8.9 (8.4-9.4)	9.0 (8.5-9.5)	0.570
C-reactive protein (mg/dL)	0.8 (0.5-2.1)	1.6 (0.6-5.2)	0.121

Data are expressed as median (interquartile range) or number (%). BMI, body mass index; CAPD, continuous ambulatory peritoneal dialysis; APD, automated peritoneal dialysis. The Pearson chi-square test was used to compare dichotomous variables, and Mann-Whitney U test was used to compare continuous variables.

### Zoonotic peritonitis were observed in pet owners

During the follow-up period, 2 cases of peritonitis caused by zoonotic organisms were noted (Table 2, S3 and S4 Tables in S1 File). These cases involved a cat owner infected with *Pasteurella dagmatis*, and a dog owner infected with *Pantoea agglomerans*. The case of *Pantoea agglomerans* infection is detailed further in a case report [15]. Notably, both patients were undergoing automated peritoneal dialysis (APD).

**Table 2:** Causative organisms of peritonitis according to pet ownership status.

Strains	Pet Owners (*n* = 24)		Non-Pet Owners (*n* = 138)		P value
	Incidence rate^†^ (95% CI)	Ratio% (Episodes)	Incidence rate^†^ (95% CI)	Ratio% (Episodes)	
G(+) bacteria	0.09 (0.04-0.18)	47.1 (8)	0.14 (0.11-0.17)	48.6 (68)	0.906
*Staphylococcus spp.*	0.03 (0.009-0.09)	17.6 (3)	0.06 (0.04-0.08)	20.0 (28)	0.818
*Staphylococcus aureus*	0.02 (0.004-0.08)	11.8 (2)	0.006 (0.002-0.02)	2.1 (3)	0.033
Coagulase-negative Staphylococci	0.01 (0.0006-0.06)	5.9 (1)	0.05 (0.03-0.07)	17.9 (25)	0.210
*Streptococcus spp.*	0.05 (0.01-0.11)	23.5 (4)	0.03 (0.02-0.05)	12.1 (17)	0.193
*Streptococcus agalactiae*	N/A	0.0 (0)	0.002 (0.0001-0.01)	0.7 (1)	0.727
Viridans streptococci	0.05 (0.01-0.11)	23. 5 (4)	0.03 (0.02-0.05)	11.4 (16)	0.158
*Enterococcus spp.*	N/A	0.0 (0)	0.01 (0.006-0.03)	5.0 (7)	0.346
*Acinetobacter spp.*	N/A	0.0 (0)	0.01 (0.004-0.02)	3.6 (5)	0.428
*Corynebacterium spp.*	N/A	0.0 (0)	0.008 (0.003-0.02)	2.9 (4)	0.480
G(-) bacteria	0.05 (0.01-0.11)	23.5 (4)	0.04 (0.03-0.07)	15.7 (22)	0.413
Enteric G(-) bacteria	0.01 (0.0006-0.06)	5.9 (1)	0.03 (0.01-0.05)	9.2 (13)	0.642
*Escherichia coli*	0.01 (0.0006-0.06)	5.9 (1)	0.02 (0.009-0.03)	6.4 (9)	0.931
*Pseudomonas aeruginosa*	N/A	0.0 (0)	0.002 (0.0001-0.01)	0.7 (1)	0.727
*Pasteurella dagmatis*	0.01 (0.0006-0.06)	5.9 (1)	N/A	0.0 (0)	0.004
*Pantoea agglomerans*	0.01 (0.0006-0.06)	5.9 (1)	N/A	0.0 (0)	0.004
*Mycobacterium tuberculosis*	N/A	0.0 (0)	0.004 (0.006-0.01)	1.4 (2)	0.620
Fungus	N/A	0.0 (0)	0.006 (0.002-0.02)	2.1 (3)	0.542
Polymicrobial infection	0.01 (0.0006-0.06)	5.9 (1)	0.02 (0.01-0.04)	7.9 (11)	0.772
Culture negative	0.05 (0.01-0.11)	23. 5 (4)	0.07 (0.05-0.10)	24.3 (34)	0.945
Total	0.20 (0.12-0.31)	100.0 (17)	0.28 (0.24-0.33)	100.0 (140)	

†Unit: events per person-year. G(+), gram-positive; G(-), gram-negative; *spp.*, species. 95% confidence interval (95% CI) of incidence rate was calculated by mid-P exact test. The Pearson chi-square test was used to compare dichotomous variables.

When examining the incidence rate of peritonitis, pet ownership (Incidence Rate = 0.20 events/person-year, 95% CI 0.12–0.31, Table 2) did not result in a higher peritonitis rate compared to patients without pets (Incidence Rate = 0.28 events/person-year, 95% CI 0.24–0.33, Table 2). Further analysis by pet type revealed similar findings: both cat owners (Incidence Rate = 0.26 events/person-year, 95% CI 0.13–0.46, S3 Table in S1 File) and dog owners (Incidence Rate = 0.15 events/person-year, 95% CI 0.06–0.29, S3 Table in S1 File) showed no significant differences in peritonitis rates compared to non-pet owners. The occurrence and causative organisms of peritonitis events for each patient are listed in S5 Table in S1 File.

Additionally, regarding prognosis following peritonitis, no significant differences were observed between patients with or without pets (S6 and S7 Tables in S1 File). Across the entire cohort, 6 peritonitis-related deaths and 16 catheter removal events were recorded during the observation period.

### Staphylococcus-associated exit-site infections according to pet ownership

When analyzing ESI events, a distinct pattern was observed in the microbiological profile of infections based on pet ownership. Among pet owners, *Staphylococcus* species were the major pathogens responsible for 5 out of 9 ESI events, more frequently observed compared to non-pet owners (55.6 vs. 16.2%, p = 0.006, Table 3). Furthermore, 4 out of 5 Staphylococcal ESI events in pet owners were in cat owners (S8 Table in S1 File).

**Table 3:** Causative organisms of exit-site infection according to pet ownership status.

Strains	Pet Owners (*n* = 24)		Non-Pet Owners (*n* = 138)		P value
	Incidence rate^†^ (95% CI)	Ratio% (Episodes)	Incidence rate^†^ (95% CI)	Ratio% (Episodes)	
G(+) bacteria	0.09 (0.04-0.18)	88.9 (8)	0.09 (0.06-0.12)	61.8 (42)	0.109
*Staphylococcus spp.*	0.06 (0.02-0.13)	55.6 (5)	0.02 (0.01-0.04)	16.2 (11)	0.006
*Staphylococcus aureus*	0.01 (0.0006-0.06)	11.1 (1)	0.01 (0.006-0.03)	10.3 (7)	0.940
Coagulase-negative Staphylococci	0.05 (0.01-0.11)	44.4 (4)	0.01 (0.003-0.02)	7.4 (5)	0.001
*Streptococcus spp.*	N/A	0.0 (0)	0.008 (0.003-0.02)	5.9 (4)	0.455
*Enterococcus spp.*	N/A	0.0 (0)	0.004 (0.006-0.01)	2.9 (2)	0.602
G(-) bacteria	0.01 (0.0006-0.06)	11.1 (1)	0.05 (0.03-0.07)	33.8 (23)	0.167
Enteric G(-) bacteria	N/A	0.0 (0)	0.002 (0.0001-0.01)	1.5 (1)	0.714
*Pseudomonas* *aeruginosa*	0.01 (0.0006-0.06)	11.1 (1)	0.03 (0.02-0.05)	25.0 (17)	0.355
Fungus	N/A	0.0 (0)	0.004 (0.006-0.01)	2.9 (2)	0.602
Unspecified (Mixed)	N/A	0.0 (0)	0.002 (0.0001-0.01)	1.5 (1)	0.714
Total	0.10 (0.05-0.19)	100.0 (9)	0.14 (0.11-0.17)	100.0 (68)	

†Unit: events per person-year. G(+), gram-positive; G(-), gram-negative; *spp.*, species. 95% confidence interval (95% CI) of incidence rate was calculated by mid-P exact test. The Pearson chi-square test was used to compare dichotomous variables.

Meanwhile, the presence of pets did not significantly affect the overall incidence rate of ESI events (Table 3). Further analysis of dog ownership and cat ownership revealed consistent results, showing no significant differences (S8 Table in S1 File). The occurrence and causative organisms of ESI events for each patient are listed in S9 Table in S1 File.

### Tunnel infection associated with pet ownership

During the follow-up period, a total of 6 TI events were observed, with 2 occurring in pet owners (Table 4). Notably, the patients with these infections were both cat owners. Expressed as proportions by group, 2.4% of non-pet owners (4 of 138), 8.3% of pet owners overall (2 of 24), and 25.0% of cat owners specifically (2 of 8) experienced TI.

**Table 4:** Clinical features of tunnel infections occurred during the observation period.

Case	Age/ Sex	Pet type	PD duration (years)	Cultured organism	Clinical Presentation	Concurrent Peritonitis	Concurrent ESI	Catheter status
1	48/ F	Cat	12.3	*Corynebacterium spp.*, Coagulase-negative Staphylococci	Tunnel tenderness	No	No	Maintained
2	37/ M	Cat	22.8	Culture negative	Tunnel route skin abrasion with discharge	No	No	Catheter reposition
3	65/ M	None	9.3	*Staphylococcus aureus*, *Streptococcus agalactiae* *Corynebacterium spp.*	Abdominal distention Tunnel tenderness	No	No	Catheter removed
4	42/ F	None	7.0	*Staphylococcus aureus*	Tunnel site pain	Yes	No	Catheter removed
5	43/ F	None	8.9	*Morganella morganii*, *Enterococcus faecalis*	Tunnel site pain and pus-like discharge	No	No	Maintained
6	54/ F	None	10.1	*Staphylococcus epidermidis*, *Candida parapsilosis*	Tunnel site culture positive	Yes	Yes	Catheter removed

PD, peritoneal dialysis; ESI, exit-site infection; M, man; F, female; *spp.*, species. Catheter removal events due to tunnel infections (TI) were defined as catheter removals occurring within 50 days after the onset of TI.

TI cases in cat owners presented with distinctive clinical features: the patient in case 1 experienced 3 additional ESI events along with the TI during the follow-up period, while the patient in case 2 had catheter reposition following the TI. Meanwhile, the other patients experienced either one additional ESI (cases 2–4) or none (cases 5 and 6). However, no catheter removal was required due to TI in pet owners, whereas TI led to catheter removal in three other patients.

## Discussion

Our study investigated PD-related infection profiles by pet ownership status, such as peritonitis, ESI, and TI. The study results showed that zoonotic peritonitis was observed during the follow-up period. Moreover, Staphylococcus-associated ESIs were more frequently observed among patients with pet ownership in this cohort. Lastly, 33% of TIs during the follow-up period were descriptively noted in pet owners, with one requiring catheter reoperation.

The most recent ISPD guideline recommends the need for additional precautions to prevent peritonitis in the presence of indoor pets [6]. Specifically, they highlight the risk of infections caused by zoonotic organisms through close contact with companion animals. Our study also identified cases of peritonitis caused by zoonotic organisms, including *Pasteurella dagmatis* and *Pantoea agglomerans*. *Pasteurella* species is a microorganism commonly colonized in cats, which can be transmitted through bites or scratches [11]. Because APD requires long external tubing and a warming plate that may attract pets, patients undergoing this modality may be more vulnerable to pet-related contamination. Consistently, within our cohort, both patients with zoonotic infections in our study were on APD. These observations suggest that awareness of potential pet-related injuries may be particularly relevant for patients with pet ownership undergoing APD [16].

In addition, Staphylococcus-associated ESIs were more frequently observed among patients with pet ownership in this cohort. Globally, including Korea, *Staphylococcus* is reported as the most common causative organism for ESIs, responsible for 30–50% of cases [17–20]. Even though coagulase-negative *Staphylococcus* species generally pose a relatively lower risk and can be managed with appropriate antibiotics according to the clinical context, *Staphylococcus aureus*, particularly methicillin-resistant strains, exhibits high virulence and antibiotic resistance and requires careful management. Accordingly, the use of prophylactic agents and the implementation of preventive strategies have been recommended to reduce the risk of Staphylococcus-associated ESIs [7].

Previously, based on data from the Korea PDOPPS study, we demonstrated that xerosis is significantly associated with an elevated risk of ESIs, with a particularly pronounced increase in the risk of *S. aureus*-associated ESIs [21]. Notably, the relative abundance of *S. aureus* within the skin microbiota of dialysis patients was higher under xerotic conditions, potentially explaining the increased susceptibility to ESIs observed with greater xerosis severity [22]. Moreover, our previous study identified pet ownership as a significant risk factor for xerosis in PD patients. Pet-related substances, including saliva and dander, have been suggested to compromise skin integrity or alter the skin microbiome of patients [23,24]. Against this background, pet ownership may be considered a contextual factor when interpreting the characteristics of Staphylococcus-associated ESIs in PD patients, based on descriptive observations from the present study.

Lastly, a proportion of TI events (33%) was descriptively observed among patients with pet ownership, particularly cat owners. Due to the limited number of events, no conclusions can be drawn regarding the relationship between pet ownership and TI.

Even so, we confirmed that pet ownership, whether of a cat or a dog, did not increase the incidence of peritonitis or ESI events among patients at this center. This finding aligns with previous findings, including recent global PDOPPS studies, that also explored the relationship between pet ownership and peritonitis [9,25]. Additionally, our findings indicate that the prognosis of peritonitis is unaffected by pet ownership.

Pet ownership offers numerous emotional benefits, potentially enhancing long-term outcomes by improving well-being and life satisfaction [26]. Emotional stability is especially crucial for dialysis patients as it can significantly enhance their quality of life and prognosis, and we have investigated this issue extensively in our studies [27,28]. Consequently, instead of restricting pet ownership, attention to hygiene and avoidance of potential injuries may be important for patients who keep pets [6].

This study has several limitations. First, despite using data from patients treated at this center over an extended period of eight years, the small number of pet owners (n = 24) and the relatively low incidence of PD-related infections limited the statistical power to detect meaningful differences, increasing the risk of a type II error. Therefore, the absence of a significant association between pet ownership and infection risk should be interpreted with caution. In addition, multivariable adjustment for potential confounders—such as diabetes, dialysis vintage, PD modality, nutritional status, and residual kidney function—was not performed due to the limited sample size and number of events, and residual confounding cannot be excluded. To provide more robust clinical evidence, an analysis involving a larger population is necessary.

Moreover, pet ownership was determined based on observations made by PD nurses during home visits. In cases where pets were not visible during these visits, there is a possibility that some instances of pet ownership may have been missed. In addition, detailed information regarding pet exposures—such as the level of contact with PD equipment, access to the dialysis room, and history of pet-related injuries—was not systematically collected. As a result, the degree of exposure to potential zoonotic pathogens could not be fully assessed. Therefore, all subgroup and pathogen-specific findings should be interpreted as exploratory.

Nevertheless, to our knowledge, our study is the first to describe PD-related infection characteristics in the context of pet ownership in Korea. With the global increase in pet ownership, including in Korea, understanding the impact of pets on PD patients is becoming more crucial than ever. Further research is needed to better assess the influence of pets on PD outcomes.

## Supporting information

S1 File Supplementary materials including Tables S1–S11. (DOCX)

## Abbreviations

APD: Automated peritoneal dialysis
BMI: Body mass index
CI: Confidence interval
ESI: Exit-site infection
ESKD: End-stage kidney disease
HD: Hemodialysis
IR: Infection rate
IRB: Institutional review board
ISPD: International Society for Peritoneal Dialysis
PD: Peritoneal dialysis
PDOPPS: Peritoneal Dialysis Outcomes and Practice Patterns Study
TI: Tunnel infection
