# Supplementary material for: Characteristics of peritoneal dialysis-related infection according to pet ownership: An 8-year single-center experience
Source: PLoS One. 2026 May 7;21(5):e0348012. doi: 10.1371/journal.pone.0348012 (PMC13152145; doi:10.1371/journal.pone.0348012)
Supplement: S1 File — (DOCX) [file pone.0348012.s001.docx]

S1 Table. Baseline characteristics of patients according to pet types

| Variables | Cat Owners  (*n* = 8) | Dog Owners  (*n* = 16) | Non-Pet Owners  (*n* = 138) | P value |
| --- | --- | --- | --- | --- |
| Demographics |  |  |  |  |
| Age (years) | 50.2 (39..9-55.6) | 45.2 (34.5-58.3) | 52.9 (44.4-61.9) | 0.273 |
| Male sex (%) | 37.5 | 43.8 | 55.8 | 0.422 |
| Dialysis vintage (month) | 164.4 (121.9-239.9) | 144.3 (61.6-185.6) | 135.3 (64.1-247.2) | 0.541 |
| BMI (kg/m^2^) | 21.5 (18.0-24.7) | 26.5 (23.5-31.3) | 24.5 (21.5-27.4) | 0.012 |
| PD type |  |  |  |  |
| CAPD (%) | 62.5 | 50.0 | 58.7 | 0.978 |
| APD (%) | 37.5 | 50.0 | 39.1 |  |
| CAPD + APD (%) | 0.0 | 0.0 | 1.4 |  |
| Hybrid (%) | 0.0 | 0.0 | 0.7 |  |
| Primary disease |  |  |  |  |
| Diabetes Mellitus (%) | 12.5 | 37.5 | 47.8 | 0.244 |
| Hypertension (%) | 50.0 | 31.3 | 34.1 |  |
| Chronic Glomerulonephritis (%) | 37.5 | 31.3 | 12.3 |  |
| Others (%) | 0.0 | 0.0 | 1.4 |  |
| Unknown (%) | 0.0 | 0.0 | 4.3 |  |
| Laboratory test |  |  |  |  |
| Hemoglobin (g/dL) | 10.5 (9.8-11.4) | 10.1 (9.2-10.9) | 10.2 (9.2-11.1) | 0.646 |
| Glucose (mg/dL) | 93.5 (86.0-102.3) | 105.5 (98.3-126.0) | 109.5 (93.0-142.3) | 0.122 |
| Albumin (g/dL) | 3.9 (3.6-4.2) | 3.9 (3.6-4.3) | 3.8 (3.4-4.1) | 0.395 |
| Total cholesterol (mg/dL) | 172.0 (144.8-199.3) | 165.0 (125.8-195.0) | 146.0 (119.8-173.0) | 0.142 |
| Triglycerides (mg/dL) | 136.0 (104.8-199.8) | 164.5 (114.5-192.8) | 124.0 (85.8-177.8) | 0.202 |
| Potassium (mEq/L) | 4.4 (4.2-4.7) | 4.5 (4.1-4.8) | 4.5 (3.9-4.9) | 0.996 |
| Phosphorus (mg/dL) | 5.4 (4.2-6.6) | 5.0 (4.2-6.4) | 4.9 (4.0-5.8) | 0.473 |
| Total calcium (mg/dL) | 9.0 (8.6-10.0) | 8.8 (8.4-9.4) | 9.0 (8.5-9.5) | 0.653 |
| C-reactive protein (mg/dL) | 0.7 (0.5-1.0) | 1.0 (0.5-4.3) | 1.6 (0.6-5.2) | 0.210 |

Data are expressed as median (interquartile range) or number (%). BMI, body mass index; CAPD, continuous ambulatory peritoneal dialysis; APD, automated peritoneal dialysis. The Pearson chi-square test was used to compare dichotomous variables, and Kruskal-Wallis test was used to compare continuous variables.

S2 Table. Pet ownership profiles in patients

| Variables | | Cat Owners  (*n* = 8) | Dog Owners  (*n* = 16) |
| --- | --- | --- | --- |
| Number of pets (%) | 1 | 50.0 | 75.0 |
|  | 2 | 25.0 | 0.0 |
|  | 3 | 0.0 | 6.3 |
|  | Unknown | 25.0 | 18.8 |
| Kept indoors or outdoors (%) | Indoors | 50.0 | 81.3 |
|  | Outdoors | 0.0 | 0.0 |
|  | Both | 25.0 | 0.0 |
|  | Unknown | 25.0 | 18.8 |

Data are expressed as number (%).

S3 Table. Causative organisms of peritonitis according to pet types

| Strains | Cat Owners  (*n* = 8) | | Dog Owners  (*n* = 16) | | Non-Pet Owners  (*n* = 138) | | P value |
| --- | --- | --- | --- | --- | --- | --- | --- |
|  | Incidence rate^†^  (95% CI) | Ratio%  (Episodes) | Incidence rate^†^  (95% CI) | Ratio%  (Episodes) | Incidence rate^†^  (95% CI) | Ratio%  (Episodes) |  |
| G(+) bacteria | 0.13  (0.05-0.29) | 50.0 (5) | 0.06  (0.02-0.17) | 42.9 (3) | 0.14  (0.11-0.17) | 48.6 (68) | 0.952 |
| *Staphylococcus spp.* | 0.03  (0.01-0.13) | 10.0 (1) | 0.04  (0.007-0.14) | 28.6 (2) | 0.06  (0.04-0.09) | 20.0 (28) | 0.622 |
| *Staphylococcus aureus* | N/A | 0.0 (0) | 0.04  (0.007-0.14) | 28.6 (2) | 0.006  (0.002-0.02) | 2.1 (5) | < 0.001 |
| Coagulase-negative Staphylococci | 0.03  (0.01-0.13) | 10.0 (1) | N/A | 0.0 (0) | 0.05  (0.03-0.07) | 17.9 (25) | 0.392 |
| *Streptococcus spp.* | 0.10  (0.03-0.25) | 40.0 (4) | N/A | 0.0 (0) | 0.03  (0.02-0.05) | 12.1 (17) | 0.025 |
| *Streptococcus agalactiae* | N/A | 0.0 (0) | N/A | 0.0 (0) | 0.002  (0.0001-0.01) | 0.7 (1) | 0.941 |
| Viridans streptococci | 0.10  (0.03-0.25) | 40.0 (4) | N/A | 0.0 (0) | 0.03  (0.02-0.05) | 11.4 (16) | 0.019 |
| *Enterococcus spp.* | N/A | 0.0 (0) | N/A | 0.0 (0) | 0.01  (0.006-0.03) | 5.0 (7) | 0.641 |
| *Acinteobacter spp.* | N/A | 0.0 (0) | N/A | 0.0 (0) | 0.01  (0.004-0.02) | 3.6 (5) | 0.731 |
| *Corynebacterium spp.* | N/A | 0.0 (0) | N/A | 0.0 (0) | 0.008  (0.003-0.02) | 2.9 (4) | 0.779 |
| G(-) bacteria | 0.08  (0.02-0.21) | 30.0 (3) | 0.02  (0.001-0.10) | 14.3 (1) | 0.04  (0.03-0.07) | 15.7 (22) | 0.495 |
| Enteric G(-) bacteria | 0.03  (0.01-0.13) | 10.0 (1) | N/A | 0.0 (0) | 0.03  (0.01-0.05) | 9.2 (13) | 0.709 |
| *Escherichia coli* | 0.03  (0.01-0.13) | 10.0 (1) | N/A | 0.0 (0) | 0.02  (0.01-0.04) | 6.4 (9) | 0.705 |
| *Pseudomonas aeruginosa* | N/A | 0.0 (0) | N/A | 0.0 (0) | 0.002  (0.0001-0.01) | 0.7 (1) | 0.941 |
| *Pasteurella dagmatis* | 0.03  (0.01-0.13) | 10.0 (1) | N/A | 0.0 (0) | N/A | 0.0 (0) | < 0.001 |
| *Pantoea agglomerans* | N/A | 0.0 (0) | 0.02  (0.001-0.10) | 14.3 (1) | N/A | 0.0 (0) | < 0.001 |
| *Mycobacterium tuberculosis* | N/A | 0.0 (0) | N/A | 0.0 (0) | 0.004  (0.006-0.01) | 1.4 (2) | 0.884 |
| Fungus | N/A | 0.0 (0) | N/A | 0.0 (0) | 0.006  (0.002-0.02) | 2.1 (3) | 0.831 |
| Polymicrobial infection | N/A | 0.0 (0) | 0.02  (0.001-0.10) | 14.3 (0) | 0.02  (0.01-0.04) | 7.9 (11) | 0.529 |
| Culture negative | 0.05  (0.01-0.17) | 20.0 (2) | 0.04  (0.007-0.14) | 28.6 (2) | 0.07  (0.05-0.10) | 24.3 (34) | 0.919 |
| Total | 0.26  (0.13-0.46) | 100.0 (10) | 0.15  (0.06-0.29) | 100.0 (7) | 0.28  (0.24-0.33) | 100.0 (140) |  |

†Unit: events per person-year. G(+), gram-positive; G(-), gram-negative; spp., species. 95% confidence interval (95% CI) of incidence rate was calculated by mid-P exact test. The Pearson chi-square test was used to compare dichotomous variables.

S4 Table. Unusual peritonitis caused by zoonotic organisms in pet owners

| Case | Age / Sex | Pet type | PD modality | Dialysis  vintage | Cultured  organism | Antibiotic  Used / Days | Route | Hospitalization  (days) |
| --- | --- | --- | --- | --- | --- | --- | --- | --- |
| 1 | 28 / F | Cat | APD | 10 months | *Pasteurella dagmatis* | Cefazolin / 9 days Ceftazidime / 21 days | IP | 3 |
| 2 | 76 / F | Dog | APD | 15 years | *Pantoea agglomerans* | Cefazolin / 2 days  Ceftazidime / 14 days | IP | 4 |

PD, peritoneal dialysis; F, female; APD, automated peritoneal dialysis; IP, intraperitoneal injection.

S5 Table. Patients who experienced peritonitis according to pet types

| Strains | Cat Owners  (*n* = 8) | | | Dog Owners  (*n* = 16) | | | | Non-Pet Owners  (*n* = 138) | | | |
| --- | --- | --- | --- | --- | --- | --- | --- | --- | --- | --- | --- |
|  | Number | Ratio% | | Number | | Ratio% | | Number | | Ratio% | |
| Patients who experienced  peritoniits | 5 | 62.5 | 4 | | 25.0 | | 62 | | 44.3 | |  |
| G(+) bacteria | 4 | 50.0 | 1 | | 6.3 | | 41 | | 29.7 | |  |
| *Staphylococcus spp.* | 1 | 12.5 | 1 | | 6.3 | | 19 | | 13.8 | |  |
| *Staphylococcus aureus* | 1 | 12.5 | 0 | | 0.0 | | 2 | | 1.4 | |  |
| Coagulase-negative Staphylococci | 1 | 12.5 | 0 | | 0.0 | | 17 | | 12.3 | |  |
| *Streptococcus spp.* | 4 | 50.0 | 0 | | 0.0 | | 12 | | 8.7 | |  |
| *Streptococcus agalactiae* | 0 | 0.0 | 0 | | 0.0 | | 1 | | 0.7 | |  |
| Viridans streptococci | 4 | 50.0 | 0 | | 0.0 | | 11 | | 8.0 | |  |
| *Enterococcus spp.* | 0 | 0.0 | 0 | | 0.0 | | 5 | | 3.6 | |  |
| *Acinetobacter spp.* | 0 | 0.0 | 0 | | 0.0 | | 3 | | 2.2 | |  |
| *Corynebacterium spp.* | 0 | 0.0 | 0 | | 0.0 | | 4 | | 2.9 | |  |
| G(-) bacteria | 3 | 37.5 | 1 | | 6.3 | | 18 | | 13.0 | |  |
| Enteric G(-) bacteria | 1 | 12.5 | 0 | | 0.0 | | 12 | | 8.7 | |  |
| *Escherichia coli* | 1 | 12.5 | 0 | | 0.0 | | 9 | | 6.5 | |  |
| *Pseudomonas aeruginosa* | 0 | 0.0 | 0 | | 0.0 | | 0 | | 0.0 | |  |
| *Pasteurella dagmatis* | 1 | 12.5 | 0 | | 0.0 | | 0 | | 0.0 | |  |
| *Pantoea agglomerans* | 0 | 0.0 | 1 | | 6.3 | | 0 | | 0.0 | |  |
| *Mycobacterium tuberculosis* | 0 | 0.0 | 0 | | 0.0 | | 2 | | 1.4 | |  |
| Fungus | 0 | 0.0 | 0 | | 0.0 | | 3 | | 2.2 | |  |
| Polymicrobial infection | 0 | 0.0 | 1 | | 6.3 | | 10 | | 7.2 | |  |
| Culture negative | 1 | 12.5 | 2 | | 12.5 | | 23 | | 16.7 | |  |

G(+), gram-positive; G(-), gram-negative.

S6 Table. Prognosis after peritonitis according to pet ownership status

| Outcomes | Pet Owners | | Non-Pet Owners | |
| --- | --- | --- | --- | --- |
|  | Episodes | Ratio% | Episodes | Ratio% |
| Cases, *N* | 17 |  | 140 |  |
| Catheter removal | 1 | 5.9 | 15 | 10.7 |
| Death | 1 | 5.9 | 5 | 3.6 |

S7 Table. Prognosis after peritonitis according to pet types

| Outcomes | Cat Owners | | Dog Owners | | Non-Pet Owners | |
| --- | --- | --- | --- | --- | --- | --- |
|  | Episodes | Ratio% | Episodes | Ratio% | Episodes | Ratio% |
| Cases, *N* | 10 |  | 7 |  | 140 |  |
| Catheter removal | 0 | 0.0 | 1 | 14.3 | 15 | 10.7 |
| Death | 0 | 0.0 | 1 | 14.3 | 5 | 3.6 |

S8 Table. Causative organisms of exit-site infection according to pet types

| Strains | Cat Owners  (*n* = 8) | | Dog Owners  (*n* = 16) | | Non-Pet Owners  (*n* = 138) | | P value |
| --- | --- | --- | --- | --- | --- | --- | --- |
|  | Incidence rate^†^  (95% CI) | Ratio%  (Episodes) | Incidence rate^†^  (95% CI) | Ratio%  (Episodes) | Incidence rate^†^  (95% CI) | Ratio%  (Episodes) |  |
| G(+) bacteria | 0.13  (0.05-0.29) | 100.0 (5) | 0.06  (0.02-0.17) | 75.0 (3) | 0.09  (0.06-0.12) | 61.8 (42) | 0.204 |
| *Staphylococcus spp.* | 0.10  (0.03-0.25) | 80.0 (4) | 0.02  (0.001-0.10) | 25.0 (1) | 0.02  (0.01-0.04) | 16.2 (11) | 0.003 |
| *Staphylococcus aureus* | N/A | 0.0 (0) | 0.02  (0.001-0.10) | 25.0 (1) | 0.01  (0.006-0.03) | 10.3 (7) | 0.473 |
| Coagulase-negative Staphylococci | 0.10  (0.03-0.25) | 80.0 (4) | N/A | 0.0 (0) | 0.01  (0.003-0.02) | 7.4 (5) | < 0.001 |
| *Streptococcus spp.* | N/A | 0.0 (0) | N/A | 0.0 (0) | 0.008  (0.003-0.02) | 5.9 (4) | 0.756 |
| *Enterococcus spp.* | N/A | 0.0 (0) | N/A | 0.0 (0) | 0.004  (0.006-0.01) | 2.9 (2) | 0.873 |
| G(-) bacteria | N/A | 0.0 (0) | 0.02  (0.001-0.10) | 25.0 (1) | 0.05  (0.03-0.07) | 33.8 (23) | 0.278 |
| Enteric G(-) bacteria | N/A | 0.0 (0) | N/A | 0.0 (0) | 0.002  (0.0001-0.01) | 1.5 (1) | 0.935 |
| *Pseudomonas aeruginosa* | N/A | 0.0 (0) | 0.02  (0.001-0.10) | 25.0 (1) | 0.03  (0.02-0.05) | 25.0 (17) | 0.442 |
| Fungus | N/A | 0.0 (0) | N/A | 0.0 (0) | 0.004  (0.006-0.01) | 2.9 (2) | 0.873 |
| Unspecified (Mixed) | N/A | 0.0 (0) | N/A | 0.0 (0) | 0.002  (0.0001-0.01) | 1.5 (1) | 0.935 |
| Total | 0.13  (0.05-0.29) | 100.0 (5) | 0.08  (0.03-0.20) | 100.0 (4) | 0.14  (0.11-0.17) | 100.0 (68) |  |

†Unit: events per person-year. G(+), gram-positive; G(-), gram-negative; spp., species. 95% confidence interval (95% CI) of incidence rate was calculated by mid-P exact test. The Pearson chi-square test was used to compare dichotomous variables.

S9 Table. Patients who experienced exit-site infections according to pet types

| Strains | Cat Owners  (*n* = 8) | | | Dog Owners  (*n* = 16) | | | | Non-Pet Owners  (*n* = 138) | | | |
| --- | --- | --- | --- | --- | --- | --- | --- | --- | --- | --- | --- |
|  | Number | Ratio% | | Number | | Ratio% | | Number | | Ratio% | |
| Patients who experienced  exit-site infection | 3 | 37.5 | 2 | | 12.5 | | 40 | | 28.6 | |  |
| G(+) bacteria | 2 | 25.0 | 2 | | 12.5 | | 34 | | 24.6 | |  |
| *Staphylococcus spp.* | 1 | 12.5 | 1 | | 6.3 | | 19 | | 13.8 | |  |
| *Staphylococcus aureus* | 0 | 0.0 | 1 | | 6.3 | | 9 | | 6.5 | |  |
| Coagulase-negative Staphylococci | 1 | 12.5 | 0 | | 0.0 | | 10 | | 7.2 | |  |
| *Streptococcus spp.* | 0 | 0.0 | 0 | | 0.0 | | 6 | | 4.3 | |  |
| *Enterococcus spp.* | 0 | 0.0 | 0 | | 0.0 | | 1 | | 0.7 | |  |
| G(-) bacteria | 2 | 25.0 | 1 | | 6.3 | | 15 | | 10.9 | |  |
| Enteric G(-) bacteria | 0 | 0.0 | 0 | | 0.0 | | 1 | | 0.7 | |  |
| *Pseudomonas aeruginosa* | 0 | 0.0 | 1 | | 6.3 | | 11 | | 8.0 | |  |
| Fungus | 0 | 0.0 | 0 | | 0.0 | | 4 | | 2.9 | |  |
| Unspecified (Mixed) | 0 | 0.0 | 0 | | 0.0 | | 1 | | 0.7 | |  |

G(+), gram-positive; G(-), gram-negative; *spp.*, species.

S10 Table. Event-level data of peritonitis episodes during the study period

| Patient No. | Pet type | Strains | Hospitalization event | Catheter removal event | Death  event |
| --- | --- | --- | --- | --- | --- |
| 001 | None | *Pseudomonas aeruginosa*, *Klebsiella pneumoniae* | Yes | Yes | No |
| 002 | None | *Staphylococcus capitis* | No | No | No |
| 002 | None | *Enterobacter cloacae*, *Streptococcus vestibularis* | Yes | No | No |
| 002 | None | *Streptococcus salivarius* | Yes | No | No |
| 006 | Dog | *Bacillus* species | Yes | No | No |
| 006 | Dog | Methicillin-resistant *Staphylococcus aureus* | Yes | No | No |
| 006 | Dog | Methicillin-resistant *Staphylococcus aureus* | No | No | No |
| 006 | Dog | Vancomycin-resistant *Enterococcus faecium*,  *Candida albicans*, *Lacticaseibacillus rhamnosus* | Yes | No | Yes |
| 009 | None | Culture negative | Yes | No | No |
| 011 | Cat | *Staphylococcus haemolyticus* | No | No | No |
| 011 | Cat | *Streptococcus sanguinis* | No | No | No |
| 011 | Cat | *Escherichia coli* | Yes | No | No |
| 012 | None | Culture negative | Yes | No | No |
| 012 | None | *Streptococcus oralis* | No | No | No |
| 012 | None | *Pseudomonas aeruginosa* | Yes | No | No |
| 013 | None | *Aspergillus fumigatus* | No | Yes | No |
| 014 | None | *Streptococcus agalactiae* | Yes | No | No |
| 014 | None | *Staphylococcus epidermidis* | Yes | No | No |
| 014 | None | *Staphylococcus haemolyticus* | No | No | No |
| 014 | None | *Corynebacterium amycolatum* | Yes | No | No |
| 014 | None | *Escherichia coli* | Yes | No | No |
| 014 | None | *Bacteroides fragilis* | No | No | No |
| 014 | None | *Staphylococcus epidermidis* | No | No | No |
| 014 | None | *Clostridium perfringens*, *Bacillus* species,  *Corneybacterium amycolatum* | Yes | No | Yes |
| 015 | None | *Corynebacterium striatum* | Yes | No | No |
| 016 | None | *Acinetobacter baumannii* | Yes | No | No |
| 016 | None | *Acinetobacter haemolyticus* | Yes | No | No |
| 016 | None | *Bacillus* species | Yes | No | No |
| 016 | None | *Neisseria* species | Yes | No | No |
| 016 | None | Culture negative | Yes | No | No |
| 016 | None | *Staphylococcus epidermidis*,  *Acinetobacter calcoaceticus* | Yes | No | No |
| 017 | None | Culture negative | Yes | No | Yes |
| 020 | None | *Bacillus* species | No | No | No |
| 020 | None | *Bacillus* species | Yes | No | No |
| 026 | None | Culture negative | Yes | No | No |
| 028 | None | *Staphylococcus capitis* | No | No | No |
| 030 | None | *Haemophilus influenzae* | Yes | No | No |
| 030 | None | *Streptococcus vestibularis* | Yes | No | No |
| 031 | Cat | Gram-negative rods | Yes | No | No |
| 031 | Cat | *Streptococcus salivarius* | Yes | No | No |
| 033 | None | Coagulase-negative Staphylococci | No | No | No |
| 033 | None | Culture negative | No | No | No |
| 034 | None | *Escherichia coli* | No | No | No |
| 034 | None | *Mycobacterium tuberculosis* | Yes | Yes | No |
| 035 | None | *Micrococcus luteus* | Yes | No | No |
| 036 | None | *Escherichia coli* | Yes | No | No |
| 038 | None | Culture negative | No | No | No |
| 038 | None | *Enterobacter cloacae* | No | No | No |
| 038 | None | Culture negative | No | No | No |
| 038 | None | Culture negative | No | No | No |
| 038 | None | *Micrococcus* species | Yes | No | No |
| 038 | None | Culture negative | No | No | No |
| 038 | None | *Bacillus* species | No | No | No |
| 039 | None | Culture negative | Yes | No | No |
| 040 | None | Culture negative | Yes | No | No |
| 041 | None | *Enterococcus faecium* | No | No | No |
| 042 | None | Culture negative | Yes | No | No |
| 045 | None | Culture negative | Yes | No | No |
| 045 | None | *Micrococcus* species | Yes | No | No |
| 045 | None | *Acinetobacter pittii* | Yes | No | No |
| 045 | None | *Mycobacterium tuberculosis* | Yes | No | Yes |
| 047 | None | *Escherichia coli* | Yes | No | No |
| 049 | None | *Staphylococcus kloosii* | No | No | No |
| 049 | None | *Staphylococcus xylosus* | No | No | No |
| 049 | None | *Enterococcus casseliflavus* | No | No | No |
| 049 | None | *Staphylococcus ureilyticus* | Yes | No | No |
| 049 | None | *Staphylococcus cohnii* | Yes | Yes | No |
| 055 | Cat | *Streptococcus oralis* | Yes | No | No |
| 056 | None | *Roseomonas gilardii* | Yes | Yes | No |
| 058 | Dog | Culture negative | Yes | Yes | No |
| 059 | None | *Staphylococcus epidermidis* | No | No | No |
| 059 | None | *Staphylococcus epidermidis* | No | No | No |
| 059 | None | *Staphylococcus epidermidis* | No | No | No |
| 060 | None | *Staphylococcus epidermidis* | Yes | No | No |
| 062 | None | *Streptococcus salivarius* | No | No | No |
| 063 | None | *Enterococcus faecalis* | Yes | No | No |
| 063 | None | *Enterococcus faecalis* | No | No | No |
| 068 | None | *Staphylococcus capitis* | No | No | No |
| 068 | None | Culture negative | No | No | No |
| 068 | None | *Escherichia coli*, *Bacteroides thetaiotaomicron* | Yes | No | No |
| 072 | Dog | Culture negative | No | No | No |
| 074 | None | *Escherichia coli* | Yes | No | No |
| 074 | None | *Acinetobacter baumannii* | Yes | Yes | No |
| 077 | None | *Staphylococcus epidermidis* | Yes | No | No |
| 078 | None | *Escherichia coli* | Yes | No | No |
| 078 | None | *Bacillus species*, *Lacticaseibacillus casei*, *Candida glabrata* | Yes | No | Yes |
| 081 | Cat | Culture negative | Yes | No | No |
| 081 | Cat | *Streptococcus mitis* | Yes | No | No |
| 081 | Cat | Culture negative | No | No | No |
| 082 | None | *Staphylococcus haemolyticus* | Yes | No | No |
| 082 | None | *Corynebacterium striatum* | Yes | Yes | No |
| 090 | None | Culture negative | Yes | No | No |
| 099 | None | *Staphylococcus warneri* | Yes | No | No |
| 107 | None | Culture negative | Yes | No | No |
| 107 | None | Culture negative | No | No | No |
| 107 | None | Culture negative | Yes | No | No |
| 107 | None | *Corynebacterium striatum*,  Methicillin-resistant *Staphylococcus arueus* | Yes | No | No |
| 108 | None | Culture negative | Yes | No | No |
| 109 | None | *Microbacterium* species | Yes | No | No |
| 109 | None | Culture negative | Yes | Yes | No |
| 110 | None | *Bacteroides thetaiotaomicron* | Yes | No | No |
| 110 | None | *Staphylococcus capitis*, *Escherichia coli* | Yes | No | No |
| 110 | None | Culture negative | No | No | No |
| 110 | None | Culture negative | Yes | No | No |
| 110 | None | *Staphylococcus epidermidis* | No | No | No |
| 110 | None | *Staphylococcus warneri* | No | No | No |
| 110 | None | Culture negative | No | No | No |
| 113 | None | *Enterococcus faecium* | Yes | No | No |
| 113 | None | *Escherichia coli* | Yes | Yes | No |
| 114 | None | Culture negative | Yes | No | No |
| 114 | None | Culture negative | Yes | No | No |
| 115 | None | Culture negative | Yes | No | No |
| 115 | None | *Streptococcus salivarius* | Yes | No | No |
| 115 | None | Culture negative | Yes | No | No |
| 118 | None | *Micrococcus* species | No | No | No |
| 118 | None | *Enterococcus faecalis* | Yes | No | No |
| 121 | None | *Streptococcus sanguinis*, *Streptococcus mitis*,  *Streptococcus oralis* | Yes | No | No |
| 121 | None | *Streptococcus mitis* | Yes | No | No |
| 122 | Dog | *Pantoea agglomerans* | Yes | No | No |
| 125 | None | Culture negative | No | No | No |
| 125 | None | Culture negative | No | No | No |
| 125 | None | *Bacteroides fragilis*, *Morganella morganii* | Yes | Yes | No |
| 129 | None | Culture negative | Yes | No | No |
| 129 | None | Methicillin-susceptible *Staphylococcus aureus* | Yes | No | No |
| 129 | None | Methicillin-susceptible *Staphylococcus aureus* | Yes | Yes | No |
| 131 | None | *Streptococcus mitis*, *Streptococcus oralis* | Yes | No | No |
| 132 | Cat | *Pasteurella dagmatis* | Yes | No | No |
| 133 | None | Coagulase-negative Staphylococci | No | No | No |
| 135 | None | *Enterococcus faecalis* | No | No | No |
| 135 | None | *Enterococcus faecalis* | No | No | No |
| 135 | None | *Staphylococcus epidermidis* | Yes | No | No |
| 136 | None | *Escherichia coli*, *Klebsiella neumoniae*,  *Streptococcus gallolyticus* | Yes | No | No |
| 136 | None | *Escherichia coli* | Yes | No | No |
| 136 | None | *Streptococcus vestibularis* | No | No | No |
| 136 | None | *Corynebacterium striatum* | Yes | No | No |
| 136 | None | *Neisseria subflava*, *Streptococcus salivarius* | Yes | No | No |
| 136 | None | *Streptococcus salivarius* | No | No | No |
| 137 | None | Gram-negative coccobacilli | Yes | No | No |
| 137 | None | *Staphylococcus haemolyticus* | Yes | No | No |
| 137 | None | Culture negative | Yes | No | No |
| 141 | None | Viridans group Streptococci | Yes | No | No |
| 141 | None | *Streptococcus vestibularis* | Yes | No | No |
| 145 | None | Culture negative | Yes | No | No |
| 145 | None | *Schaalia odontolytica* | No | No | No |
| 145 | None | Viridans group Streptococci | Yes | No | No |
| 145 | None | *Streptococcus mitis*, *Streptococcus oralis* | Yes | No | No |
| 145 | None | *Escherichia coli* | Yes | No | No |
| 145 | None | Culture negative | Yes | No | No |
| 145 | None | *Streptococcus salivarius* | Yes | No | No |
| 147 | None | *Staphylococcus epidermidis* | Yes | No | No |
| 149 | None | *Staphylococcus capitis* | Yes | No | No |
| 149 | None | *Candida albicans* | Yes | No | Yes |
| 151 | None | *Acinetobacter baumannii* | Yes | Yes | No |
| 153 | None | *Streptococcus parasanguinis* | Yes | Yes | No |
| 154 | None | *Candida parapsilosis* | Yes | Yes | No |
| 156 | None | *Micrococcus* species | Yes | No | No |
| 162 | None | Methicillin-susceptible *Staphylococcus aureus* | Yes | Yes | No |

S11 Table. Event-level data of exit-site infection episodes during the study period

| Patient No. | Pet type | Strains |
| --- | --- | --- |
| 001 | None | *Klebsiella pneumoniae* |
| 002 | None | Coryneform gram-positive rods |
| 006 | Dog | Methicillin-susceptible *Staphylococcus aureus* |
| 006 | Dog | *Pseudomonas aeruginosa* |
| 009 | None | Coryneform gram-positive rods |
| 011 | Cat | Coagulase-negative Staphylococci |
| 011 | Cat | Coagulase-negative Staphylococci |
| 011 | Cat | Coagulase-negative Staphylococci |
| 012 | None | *Pseudomonas aeruginosa* |
| 013 | None | *Cutibacterium acnes* |
| 013 | None | *Neisseria* species |
| 013 | None | *Staphylococcus simulans* |
| 013 | None | *Corynebacterium striatum* |
| 014 | None | *Enterococcus faecalis* |
| 020 | None | *Pseudomonas aeruginosa* |
| 020 | None | *Pseudomonas aeruginosa* |
| 021 | None | Coryneform gram-positive rods |
| 021 | None | *Serratia marcenscens* |
| 021 | None | *Pseudomonas aeruginosa* |
| 031 | Cat | Coagulase-negative Staphylococci |
| 035 | None | Coryneform gram-positive rods |
| 038 | None | *Serratia marcenscens* |
| 042 | None | Methicillin-susceptible *Staphylococcus aureus* |
| 045 | None | Coryneform gram-positive rods |
| 049 | None | *Pseudomonas aeruginosa* |
| 049 | None | Coagulase-negative Staphylococci |
| 052 | None | *Pseudomonas aeruginosa* |
| 060 | None | Methicillin-susceptible *Staphylococcus aureus* |
| 062 | None | Coagulase-negative Staphylococci |
| 074 | None | Methicillin-susceptible *Staphylococcus aureus* |
| 074 | None | Coryneform gram-positive rods |
| 078 | None | *Pseudomonas aeruginosa* |
| 081 | Cat | *Corynebacterium striatum* |
| 082 | None | *Corynebacterium striatum* |
| 082 | None | Yeast form cells |
| 082 | None | Methicillin-resistant *Staphylococcus aureus* |
| 082 | None | Coryneform gram-positive rods |
| 083 | Dog | Coryneform gram-positive rods |
| 083 | Dog | Coryneform gram-positive rods |
| 088 | None | *Corynebacterium striatum* |
| 088 | None | *Streptococcus agalactiae* |
| 088 | None | *Corynebacterium striatum* |
| 088 | None | *Streptococcus agalactiae* |
| 088 | None | *Streptococcus agalactiae* |
| 088 | None | *Streptococcus agalactiae* |
| 088 | None | Coryneform gram-positive rods |
| 095 | None | Coryneform gram-positive rods |
| 095 | None | Coryneform gram-positive rods |
| 104 | None | *Pseudomonas aeruginosa* |
| 104 | None | *Pseudomonas aeruginosa* |
| 107 | None | *Pseudomonas aeruginosa* |
| 107 | None | *Pseudomonas aeruginosa* |
| 107 | None | *Acinetobacter baumannii* |
| 113 | None | Methicillin-susceptible *Staphylococcus aureus* |
| 114 | None | *Staphylococcus epidermidis* |
| 117 | None | Coryneform gram-positive rods |
| 118 | None | *Enterococcus faecalis* |
| 118 | None | *Neisseria* species |
| 118 | None | *Pseudomonas aeruginosa* |
| 118 | None | *Vagococcus fluvialis* |
| 121 | None | *Clostridium subterminale* |
| 127 | None | Coryneform gram-positive rods |
| 128 | None | *Pseudomonas aeruginosa* |
| 129 | None | *Corynebacterium striatum* |
| 131 | None | Coryneform gram-positive rods |
| 131 | None | *Pseudomonas aeruginosa* |
| 133 | None | Penicillin-susceptible *Staphylococcus aureus* |
| 133 | None | unspecified |
| 135 | None | Coryneform gram-positive rods |
| 136 | None | Coryneform gram-positive rods |
| 141 | None | Coryneform gram-positive rods |
| 145 | None | *Pseudomonas aeruginosa* |
| 145 | None | *Pseudomonas aeruginosa* |
| 145 | None | *Pseudomonas aeruginosa* |
| 149 | None | *Candida albicans* |
| 154 | None | *Staphylococcus epidermidis* |
| 160 | None | Coryneform gram-positive rods |
